# An Unusual Presentation of Osteochondroma in a Sexagenarian

**DOI:** 10.7759/cureus.1868

**Published:** 2017-11-21

**Authors:** MN Baig, Rajnita Auckloo, Usman Baig, S.R. Kearns

**Affiliations:** 1 Trauma & Orthopaedics, Galway University Hospital; 2 Medicine, Quaid-E-Azam Medical College, Bahawalpur.

**Keywords:** osteochondroma

## Abstract

Osteochondroma is the most commonly-found benign bone tumour. It is a benign, cartilaginous-capped bony projection. They are usually present on the bony surfaces of the long bones in adolescents and young adults. The risk of malignant transformation is <1% with solitary osteochondroma. We present a rare case of an osteochondroma in a patient with advanced age and an unusual location.

## Introduction

Osteochondromas make up 20% to 50% of benign tumours and account for approximately 10% of all bone tumours [1]. It can present as a solitary lesion or as multiple osteochondromas. The solitary kind is commonly seen in 85% of individuals with this condition. The common age of occurrence is childhood or adolescence [2-3]. It has a high incidence in long limb bones, particularly the lower limb long bones (tibia, 43%; femur, 30%; and humerus, 26%) [4-5]. They are rarely discovered in the spine, skull, hands or feet.

Osteochondromas usually present as painless, palpable, slow-growing masses. They rarely cause numbness, weakness or changes in the colour of the skin. An osteochondroma can be investigated using x-rays, computed tomography, and magnetic resonance imaging (MRI). MRI is a safe and elaborate method of investigation for a detailed evaluation of the osteochondroma and its adjacent structures.

## Case presentation

A 66-year-old woman presented to our clinic with complaints of pain on the plantar surface of the medial aspect of her left foot for the past one year. She felt a palpable mass which has been gradually and slowly increasing in size and causing pain, especially when she walks. She said the pain was bearable at the start but for the past few months, the pain has made walking increasingly difficult.

**Figure 1:**
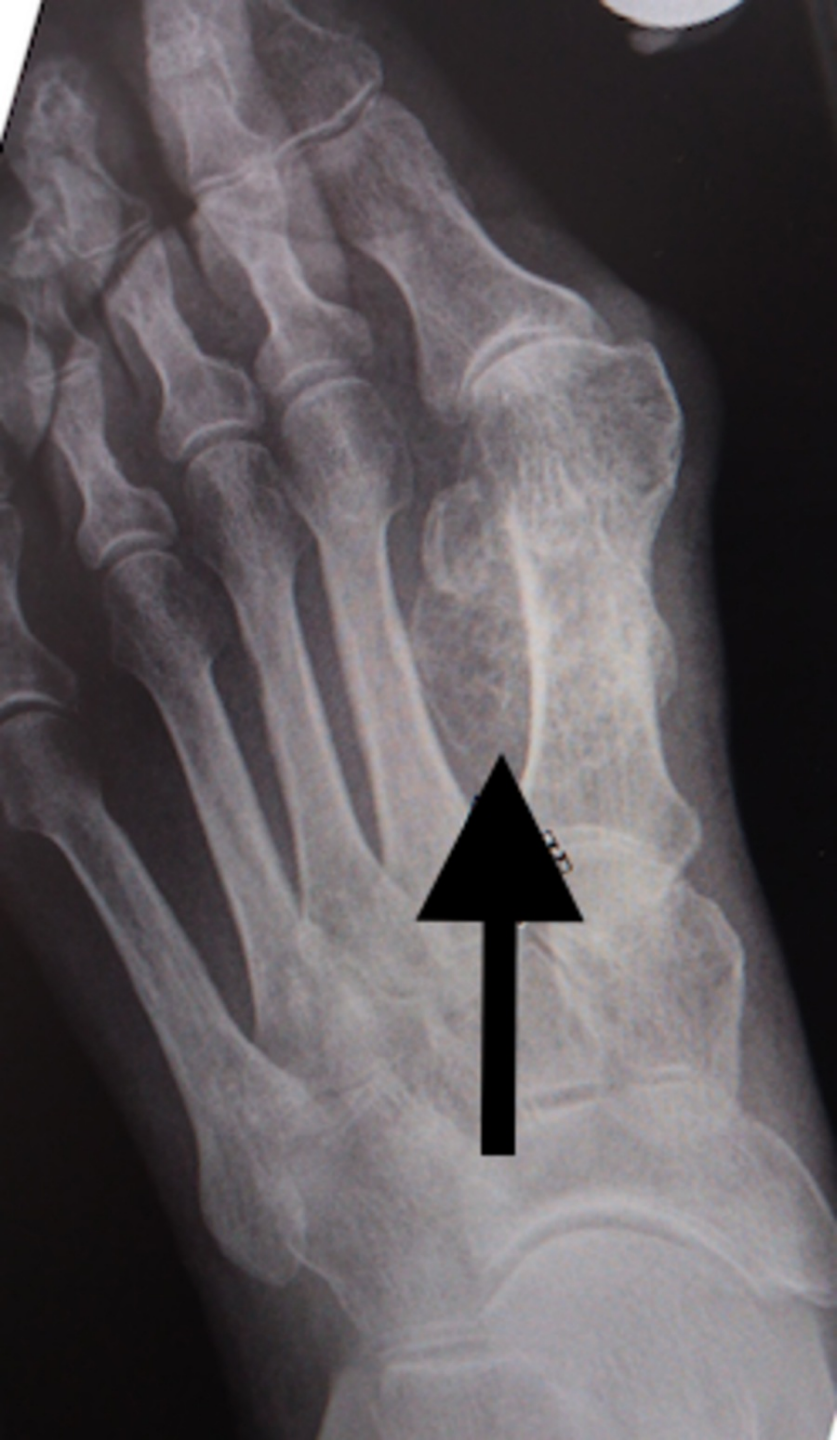
X-ray of osteochondroma. Arrow showing the osteochondroma on plantar surface of 1st metatarsal.

On physical examination, she had a palpable mass over the head of the first metatarsal on the plantar aspect. It was hard, non-mobile, mildly tender, and covered in the callused skin but without ulceration. The results of the neurovascular examination of the foot were normal. The x-ray showed a bony growth on the plantar surface of the first metatarsal (Figure 1).

We discussed with her the treatment options, including the surgical option, with its risks and complications. Given her general health and willingness to proceed, we planned for surgical exploration and excision of the mass under general anaesthesia.

**Figure 2:**
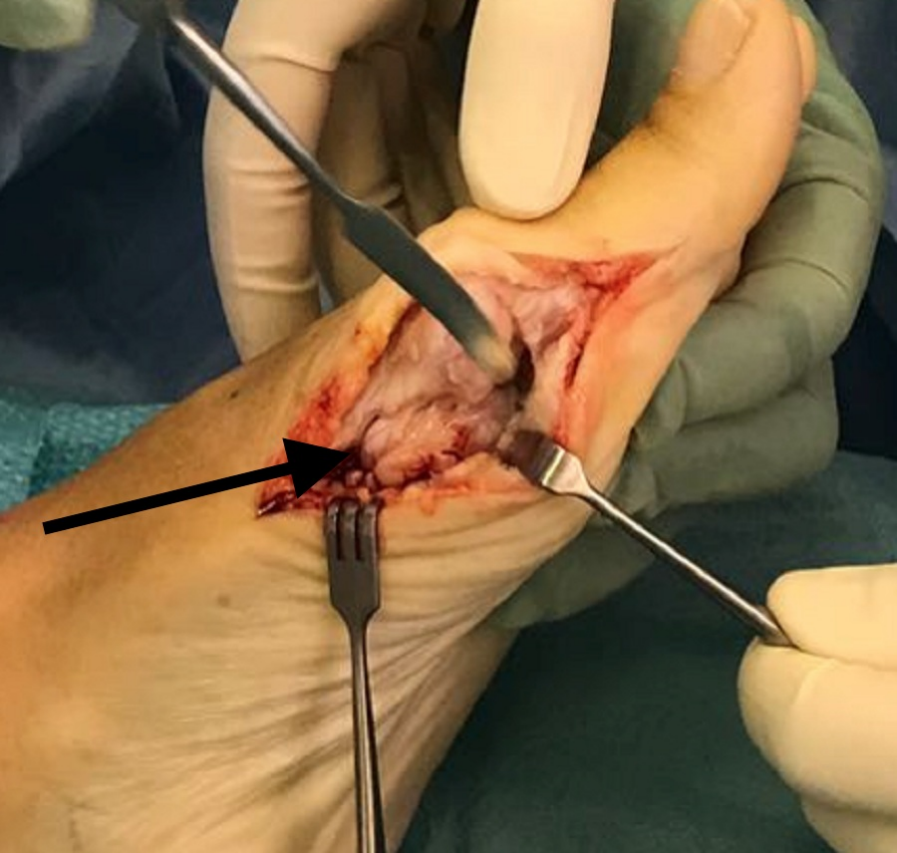
Osteochondroma. Arrow showing osteochondroma.

**Figure 3:**
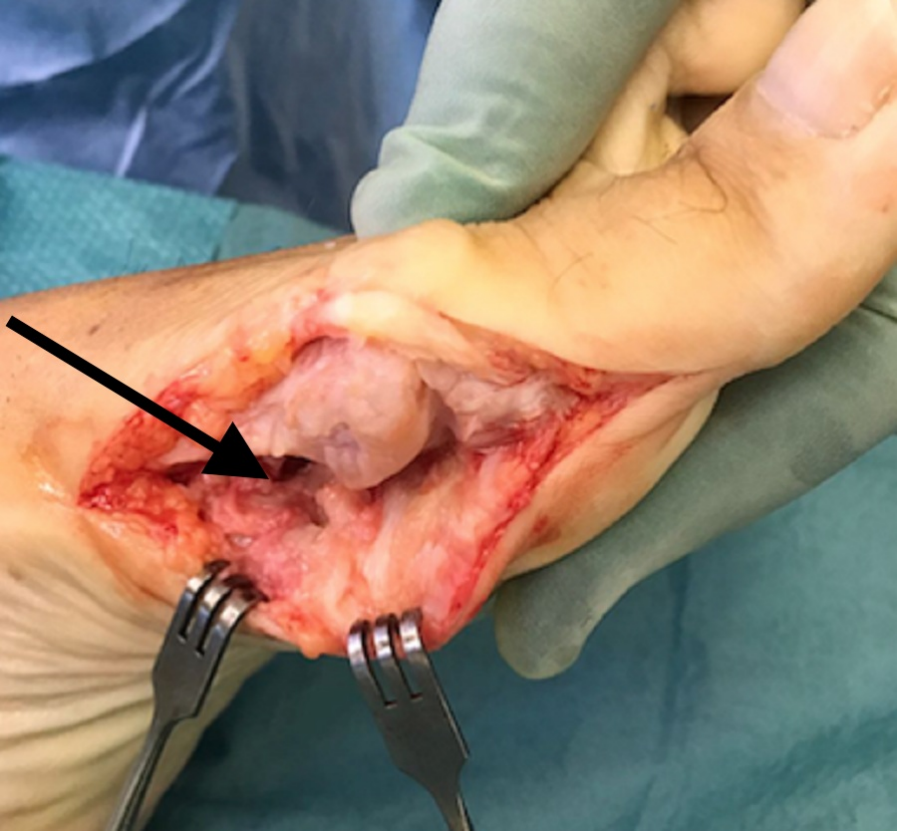
Excision of osteochondroma. Arrow showing the excision of the osteochondroma on the plantar surface.

**Figure 4:**
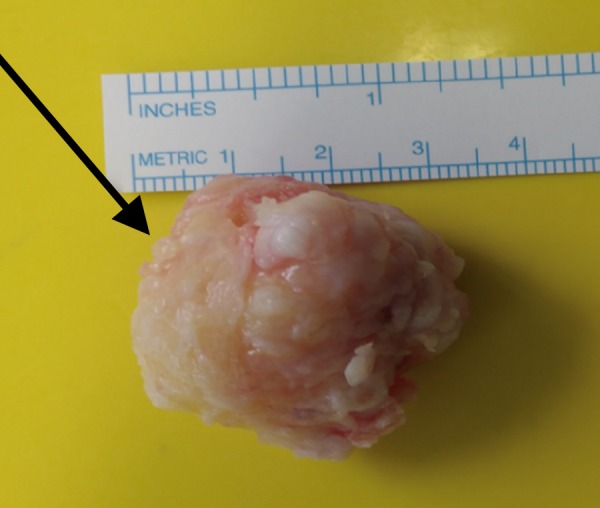
In vitro osteochondroma. Arrow showing osteochondroma of approximately 3.5 × 3 cm

**Figure 5:**
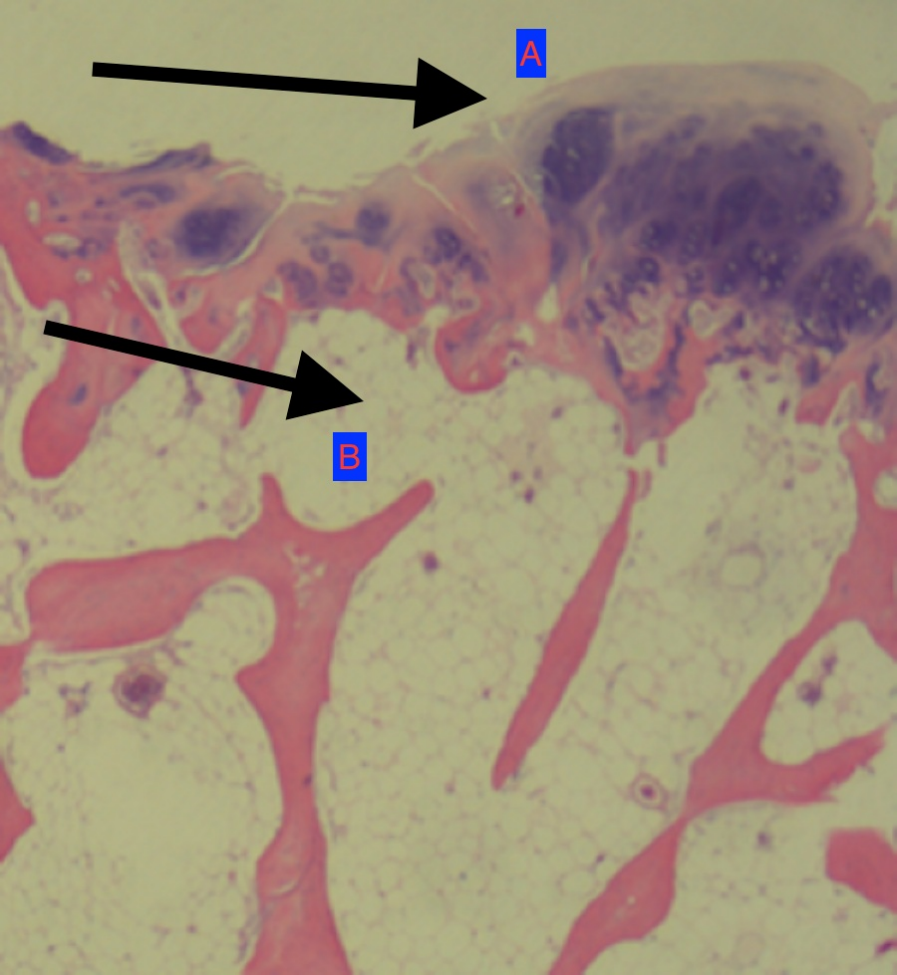
Histology slide picture. Arrows showing: (A) cartilaginous cap; (B) trabecular bone.

She was given general anaesthesia and placed in the supine position. We made a medial 5 cm incision over the left first metatarsal to gain access to the underlying structures. Once the underlying tissue was dissected, the lesion was revealed as a sessile mass over the head and neck of the plantar surface of the first metatarsal (Figure 2). We excised the mass using an osteotome (Figure 3). We cleaned the site and performed closure in layers. The mass was sent for a histopathology examination, which confirmed trabecular bone with fibrocartilaginous cap, indicative of osteochondroma (Figure 4, Figure 5).

## Discussion

Osteochondromas usually present during the patients' first three decades of life with no predilection for sex [6]. Symptoms depend on the osteochondroma’s size and location; most cases are asymptomatic and painless [7-8]. If symptoms manifest, they are usually characterized by bulging, swelling, and pain with mechanical compression on adjacent anatomical structures. Treatment is usually limited to observation if no symptoms are present [6]. Surgical removal is indicated in the presence of pain, neurovascular compromise, or if the lesion is restricting joint movement. The diagnosis is confirmed by biopsy of the specimen, and excision is usually the permanent solution. If recurrence occurs or the cartilaginous cap of the biopsy is >2 cm, the clinician should suspect a malignant transformation [9].

Our case had two distinctive features. First, our patient was 66 years old, which is a very unusual age of presentation for osteochondroma. Secondly, the osteochondroma was located at the base of the first metatarsal bone rather than the classical long bone location.

## Conclusions

Osteochondroma is the most common benign tumour in patients in their second or third decades of life, but we should always be aware of its capability for an unusual presentation, as this case illustrates.
